# Sanicula—Clinical Verifications

**Published:** 1893-03

**Authors:** J. G. Gundlach

**Affiliations:** Spokane, Wash.


### SANICULA—CLINICAL VERIFICATIONS.

J. G. Gundlach, M. D., Spokane, Wash.

Case I.—Mr. H., attorney, German, leuco-phlegmatic temperament. Had been suffering some time from the following symptoms: passes frequently large quantities of pale, colorless urine, S. G. 1,000, both day and night; worse in the daytime. Small of the back feels weak and aches; feels as though all tired out or exhausted in that region; better from pressure.

Headache (not defined).

Bad taste in the mouth. Not much appetite. Very thirsty; drinks very large drinks; wants to drink all the time.

Feet cold and damp; sweat smells bad.

The above gentleman had been to see two doctors before coming to my office, an allopath and our biochemic friend also, but neither gave him help. Remembering well my own as well as the others' proving of Sanicula, I felt sure that remedy would help this case, which it did at once, and now a year has passed and the trouble not returned.

Case II.—I. R. S., printer, aged forty. Has been suffering for some weeks, the results of over-work, with a dull pain in the forehead over the eyes; feels as if the eyes were being drawn back into the head. All made worse from being in the warm or close room and of application of the mind. Better from being in the open air.

Mind wanders when trying to apply it. Can't keep at any one thing in the office any length of time. Hardly gets started at one thing before he drops it and picks up another. When at work in the office can't content himself, and though he is proprietor and has work that must be done, yet for the most trifling reason will drop it and run out.

No appetite; bad taste, tongue coated, white, worse in the mornings; dry mouth, no thirst. He is in great distress, fears he is losing his reason, was sure he would if not helped soon. Had a spell like this some years ago, and had to stop work for a long time.

Mr. S. had come to the office in a hurry once before this, not giving me time to take the case as I ought. I gave him Puls., but did not help any; after taking the case as given above, with some study I thought Sanicula was the best, and ought to at least help him. I gave it in the 10m. It cured the case at once. Some three months have passed, and Mr. S. is still at work and all O. K. This is the first time I have had to verify these mental symptoms.

CASE III.—Mrs. H., age forty-five, chilly all the time, mingled with flushes of heat.

Chills are worse from moving around, even turning in bed; better from external warmth. These chills come at irregular times; may come on at night in bed, spreading from below up. During chill wants to be covered; when the heat comes wants the cover off.

Pains and aching in the limbs; feels sore and bruised, both flesh and bones; can't place the hand behind or on her head from pains in the shoulder.

Head feels dull and heavy.

Warmth feels good to the pains in the body but not to the head, which wants the cool; can't stand heat of stove about the head. Has bad taste in the mouth, nothing tastes good; desires sour things; some thirst with the fever; urine dark and scanty.

The above case I had given several remedies to as this condition had been going on for some weeks, the woman living in the country, but did not seem to hit it until I gave Sanicula$^{10m}$, which cured the case.

